# Imputation by the mean score should be avoided when validating a Patient Reported Outcomes questionnaire by a Rasch model in presence of informative missing data

**DOI:** 10.1186/1471-2288-11-105

**Published:** 2011-07-14

**Authors:** Jean-Benoit Hardouin, Ronán Conroy, Véronique Sébille

**Affiliations:** 1 EA 4275 "Biostatistics, Clinical Research and Subjective Measures in Health Sciences", Faculties of Medicine and Pharmaceutical Sciences, University of Nantes, 1 rue Gaston Veil, BP 53508, 44035 Nantes Cedex 1, Nantes, France; 2 Biostatistics Platform, Clinical Research Unit, University Hospital of Nantes, Nantes, France; 3 Division of Population Health Sciences, Royal College of Surgeons in Ireland, Dublin, Ireland

## Abstract

**Background:**

Nowadays, more and more clinical scales consisting in responses given by the patients to some items (Patient Reported Outcomes - PRO), are validated with models based on Item Response Theory, and more specifically, with a Rasch model. In the validation sample, presence of missing data is frequent. The aim of this paper is to compare sixteen methods for handling the missing data (mainly based on simple imputation) in the context of psychometric validation of PRO by a Rasch model. The main indexes used for validation by a Rasch model are compared.

**Methods:**

A simulation study was performed allowing to consider several cases, notably the possibility for the missing values to be informative or not and the rate of missing data.

**Results:**

Several imputations methods produce bias on psychometrical indexes (generally, the imputation methods artificially improve the psychometric qualities of the scale). In particular, this is the case with the method based on the Personal Mean Score (PMS) which is the most commonly used imputation method in practice.

**Conclusions:**

Several imputation methods should be avoided, in particular PMS imputation. From a general point of view, it is important to use an imputation method that considers both the ability of the patient (measured for example by his/her score), and the difficulty of the item (measured for example by its rate of favourable responses). Another recommendation is to always consider the addition of a random process in the imputation method, because such a process allows reducing the bias. Last, the analysis realized without imputation of the missing data (available case analyses) is an interesting alternative to the simple imputation in this context.

## Background

Patient Reported Outcomes (PRO) nowadays are commonly encountered in clinical research to take into account important unobservable characteristics. They are used for evaluating endpoints that cannot be directly observed and measured, such as Health Related Quality of Life (HR-QoL), anxiety, depressive symptoms, fatigue, addictive behaviors... Usually, patients respond to a questionnaire containing several items, with binary or ordinal responses, and the responses are often combined to give scores. The idea of clinical research is usually to compare two or more groups of patients on different outcomes that can be, for instance, PRO.

Two main types of analysis can be used to handle such data: Classical Test Theory (CTT) and Item Response Theory (IRT). In CTT, the observed scores are assumed to be a good representation of the "true" score. An alternative analysis consists in using IRT models, in which the responses to the items are modelled as a function of a latent variable. This variable is considered to be the ability measured by the questionnaire (e.g. Health Related Quality of Life, anxiety...). Among the IRT models, the Rasch model [1] is the most popular, when all the items have dichotomous responses. Indeed, this model is the most simple one, and allows the derivation of a scale with interesting psychometrical properties. In particular, it is possible to show that the estimations of the latent trait with this model are independant of the retained items. This property of specific objectivity allows the derivation of comparable measures of the latent trait with different versions of the questionnaire (for example, short or long version, with or without missing values....). As a consequence, there are compelling arguments when validating a scale, to retain only those items which show a good fit to a Rasch model [1].

Several indexes allow testing the fit of the Rasch model. As for all the models of IRT, the Rasch model relies on three fundamendal assumptions: undimensionality, local independence and monotonicity. The check of these assumptions can be realized using Loevinger's H coefficients [2,3], and in particular, the scalability coefficient H. More specifically, the fit of the Rasch model can also be considered. Among the fit tests that have been proposed, the *Q*1 test [4] is one of the most popular. However, the study of the fit of the Rasch model can only be considered if the parameters of this model are unbiased (parameters characterizing the items, and the parameters of the distribution of the latent variable, since only global measure on the sample will be generally used in clinical research). Last, the fiability of the measure of the latent trait by an IRT model can be evaluated by the Personal Separation Index (PSI) [5]. This index is close, in its interpretation, to the Cronbach's alpha [6], which is a well-known index of reliability in CTT. In the framework of PRO, it is frequent to have a non negligible rate of missing data, which are often non ignorable, because there might be a link between the measured latent variable and the probability of missingness of a response: for instance, patients with worse levels on the latent variable are more likely to have missing responses than other patients [7]. For example, in the case of HR-QoL, patients with a poor quality of life might be too tired to respond to a question or to achieve their questionnaire. This phenomenon can differently influence all the items: some items can be more affected by a large rate of missing data, such as items that deal with a topic that might be difficult to express for the patient. As a consequence, the dataset might contain more information on the patients with a good level on the latent trait, as compared to patients with a poor level, introducing bias into the subsequent analysis.

For this reason, it is important to take into account the occurence of missing data and the possibility of an underlying mecanism of missingness when analysing the dataset. Many authors suggest to replace the missing data by the most probable result: this process is called single (or simple) or multiple imputation [8]. Data are then analysed using these imputed values. Several methods have been proposed to impute missing responses to items, depending on assumptions made on the missing data mechanism. The most popular method for PRO consists in imputing a missing value by the mean response of the patient to the other items. Such a method is clearly recommended in scoring manuals of widely used questionnaires such as SF-36 and QLQ-C30 for instance [9-11]). However, it is well-known that this type of method might be inadequate [12-14], especially when the rate of missing data is high [15].

Nevertheless, such simple imputation methods have been rarely compared in the framework of psychometric validation of PRO questionnaires, especially when an analysis by IRT is planned. Among the few papers on this topic, [16] and [17] compared only a small number of methods, for bias in the estimation of Cronbach's alpha and Loevinger's H coefficient. Sijtsma and van der Ark [17] also considered the fit of the Rasch model. However, the problem of the potential bias on the estimation of the parameters of this model is more important to consider in the first place, because the fit cannot be correctly evaluated with biased parameters.

These two papers focused only on a small number of methods. Moreover, their finding are difficult to compare because different methodologies were used to simulate the missing data. Furthermore, the impact of the imputation methods on the bias in the parameters of parametric IRT models remains unknown. We therefore evaluated the impact of sixteen different methods for handling missing values in the framework of the Rasch model on (i) the bias of commonly used indices for evaluating the fundamental assumptions of IRT (Loevinger's H coefficient), (ii) the bias on the estimated parameters of the Rasch model, (iii) the bias on a fit test statistic, (iv) the bias on the measure of the fiability of the estimation of the latent trait (PSI). These parameters were chosen because they are the most important parameters for validating a Rasch model.

All these investigations were carried out using a simulation study. Such studies can contribute to give more insight from what is known from statistical theory that often provides asymptotic results. Indeed, simulations can be used to reflect real-life situations encountered in practice that can be of interest to applied researchers (various sample sizes, number of items...). Furthermore, simulation studies can help assessing the suitability and precision of different statistical models and in particular the bias in the parameter estimates in relation to a known simulated truth.

We performed a simulation study to evaluate the bias on these parameters or indices, according to the chosen method for handling missing values, the rate of missing values, and whether the missing data were ignorable or not.

## Methods

### Notation

Let

• *X_nj _*be the dichotomous variable representing the response of the *n*th individual (*n *= 1...*N*) to the *j*th item (*j *= 1...*J*) and *x_nj _*its realization [*x_nj _*= 0 denotes the more negative response to the *j*th item and *x_nj _*= 1 the positive response]

• *D_nj _*be a dummy variable taking the value 1 if *x_nj _*is observed and 0 otherwise and *d_nj _*its realization.

• *O_n _*be the set of observed responses for the *n*th individual

• *M_j _*be the set of observed responses for the *j*th item

•  be the number of observed responses for the *n*th individual

•  be the number of observed responses for the *j*th item

•  be the score of the *n*th individual (number of positive non-missing responses)

•  be the number of positive non-missing responses to the *j*th item

•  be the possibly imputed value used in the analysis for *x_nj _*(note that  if *d_nj _*= 1)

### Simulation design with (non)informative missing data

Item Response Theory (IRT) [18] is a set of models that allows measuring a latent variable Θ that influences the responses to the items. Three assumptions govern these models:

• Unidimensionality: only one latent trait influences the responses to all the items,

• Local Independence: for a given individual, the responses to the items are independent,

• Monotonicity: the probability of giving a positive response to a given item does not decrease with the latent variable.

Θ is usually considered as a random variable and θ*_n _*represents the latent trait of the *n*th patient. For each patient, the probability of responding to each item is computed according to a specific IRT model, the Rasch model [1]:(1)

where *x_nj _*= 0 for a negative response and *x_nj _*= 1 for a positive response. *δ_j _*is named the difficulty parameter of the *j*th item, because the higher its value, the lower the probability of positive response. We consider the latent variable as a random variable following a normal distribution with unknown parameters *μ *and *σ*^2^. This implies that the sample is representative of the underlying population. Using the Local Independence assumption underlying Item Response Theory (IRT), the marginal likelihood is expressed as(2)

with *G*(Θ*/μ*, *σ*^2^) the normal distribution function. Note that the Rasch model can be considered as a Generalized Linear Mixed Model with a logistic function as link function.

We estimate *δ_j_*(*j *= 1, ..., *J*), *μ *and *σ*^2 ^by maximizing this marginal likelihood [1]. The integral can be approximated with Gauss-Hermite quadratures. An identifiability constraint must be defined, and generally,  is used, but  can also be used. Let  is an estimable parameter, meaning that its estimation is independent of the chosen identifiability constraint. In the present paper, the chosen indentifiability constraint is  and consequently, a bias on the *ν *parameter represents a global bias on the *δ_j _*parameters.

Three missing data mechanisms have been described by Rubin [19]: missing completely at random (MCAR), missing at random (MAR), and missing not at random (MNAR). For instance, in case of a self-reported HR-QoL questionnaire, data can be considered MCAR if the probability of missing data (missing response on one or more items for instance) is independent of the patient's HR-QoL. Data will be considered MAR if the probability of missing data may depend on covariates describing the patients or on items characteristics [13,17]. In contrast, data will be considered MNAR if the probability of missing data depends on the patient's (unobserved) HR-QoL.

Data are simulated according to these three mechanisms, following a methodology already proposed by Sébille et al. [20] and close to the one used by Holman and Glas [21] for exploring ignorability of the missing data. More precisly, a latent variable noted *ξ *is used, corresponding to non-response propensity which represents the tendency of non-response, which varies between individuals. This latent variable may be influenced by the value of the patient's latent trait Θ (HR-QoL, fatigue,...) and may thus involve a non-ignorable non response framework corresponding to MNAR data. To simulate the missing values, we assume that each patient has a non-response propensity to each item represented by the latent variable *ξ*. The realization of *ξ *for the *n*th individual is denoted *ξ_n_*.

Let *ρ *= *Corr*(Θ, *ξ*), *w *a dummy variable (coded 0 or 1) representing the link between the presence of missing data and the difficulty of the items (*δ_j_*, *j *= 1...*J*), *π *be the expected rate of missing values for each item and *π_n _*be the probability for the *n*th patient to have a missing value to each item. This probability is assumed to have a lower bound equal to 1% and to be centred on *π*.(3)

According to the value of *ρ *and *w*, different missing data mechanisms could be simulated: for *ρ *= 0 and *w *= 0, the missing data will be MCAR, for *ρ *= 0 and *w *= 1, they are MAR, and for *ρ *≠ 0, the missing data are considered as MNAR. We assume that a patient with a low level on the latent trait (low level of HR-QoL for instance) has a higher propensity to fail to respond to the items, so *ρ *is assumed to be less than or equal to 0.

Data were simulated with three different values for *ρ*: *ρ *= 0 (MCAR or MAR data according to the value of *w*), *ρ *= -0.4 (MNAR data with low level of informativity of the missing data) and *ρ *= -0.9 (MNAR data with high level of informativity of the missing data).

A thousand replications were simulated, each with 500 individuals. Five items were used and the difficulty parameters were fixed to -1, -.5, 0, .5 and 1. The values of *θ_n _*and *ξ_n _*were drawn from a standardized normal distribution. Consequently, in all the simulations, . Three values have been considered for *π*: 10%, 20% and 30%.

We first simulated complete datasets, then created missing values by the process described above.

### Methods for handling missing data in the framework of IRT

#### No imputation - NOIMP

NOIMP is not an imputation method. It consists in treating all observed data. This method is often referred to as "available case analysis".

#### Listwise Deletion - LD

LD is not an imputation method either [17]. It consists in omitting the individuals with one or more missing values. This method is often referred to as "complete case analysis".

#### Worst case - WORST

WORST is a method which consists in substituting the "worst" results to all the missing data. Often, the more negative result is coded 0 (negative response), thus:(4)

#### Personal Mean Score - PMS and PMS-R

One of the most commonly used methods of imputation in PRO is the Personal Mean Score (PMS) method which involves imputing a missing value using the average score of the individual on the observed responses (rounded to the nearest integer) [16,17]. This method is used for example for the SF36, which is one of the most popular generic questionnaires of HR-QoL [10,11] or for the QLQ-C30 [9] which is a questionnaire of HR-QoL in Oncology.(5)

In the PMS-R method,  is randomly drawn from a Bernoulli distribution with parameter .

#### Item Mean Score - IMS and IMS-R

This method consists in imputing a missing value with the item mean score (rounded to the nearest integer) [16].(6)

In the IMS-R method,  is randomly drawn from a Bernoulli distribution with parameter .

#### Corrected Item Mean - CIM and CIM-R

PMS only takes into account the ability of the individual, and IMS only takes into account the difficulty of the item. The Corrected Item Mean method is a combination of these two methods: the item mean score is weighted by the personal mean score of the individual [16].(7)

In the CIM-R method,  is randomly drawn from a Bernoulli distribution with parameter .

#### Item Correlation substitution - ICS

This method has two steps: (i) searching for the more correlated item to each item, (ii) if the response of the *n*th individual to the *j*th item is missing, we replace it by the response of this individual to the most correlated item to the *j*th item [16].(8)

with(9)

with *X_j _*the variable representing the responses to the *j*th item (*j *= 1...*J*).

#### Logistic model - LOG and LOG-R

This method consists in fitting a logistic model to each item with missing values, with the other items as covariates [22]. A stepwise selection procedure is subsequently used to iteratively select the items that are significantly related to the missing item, as assessed by the likelihood ratio test.

That is, for an item *j *with missing values, the following final model is fitted with the items, assuming items *k*, *k *∈ *K *have been selected with the stepwise procedure (*K *is the set of the indices of the selected items, *j ∉ K*):(10)

Where *p_nj _*= *P *(*X_nj _*= 1) and .

In the LOG method,  is obtained by rounding the obtained probability, and in the LOG-R method,  is randomly drawn from a Bernoulli distribution using this probability as its parameter.

#### Mokken model - MOK

The imputation by the Mokken model [16,23] consists in substituting the missing data by the most probable values in order to obtain a responses pattern which produces the fewer Guttman errors as possible (a Guttman error is produced when an individual negatively responds to a given item, and positively responds to a more difficult item). For example, if a large proportion of the sample endorses item A and only a small proportion endorses item B, it is consider inconsistentto have an individual who endorses item B, but not item A.

If the items are ordinated from the most prevalent item to the least prevalent, a coherent vector of responses for a given individual is composed of 1s then of 0s, for example (1,1,1,0,0) or (1,0,0,0,0). The algorithm used for imputation is described here:

1. The items are sorted as a function of the number of positive responses to each of them, from the easiest item (item with the largest amount of positive responses) to the most difficult one.

2. For every missing data the following five rules are applied:

(a) If a positive response follows the missing response, impute the value 1.

(b) If not, then if a negative response precedes the missing response, impute the value 0.

(c) If not, then define *a*_00 _as the number of negative responses preceding a missing response, and *a*_01 _as the number of positive responses preceding a missing response. If *a*_00 _≥ *a*_01 _impute the value 0.

(d) If not, then define *a*_10 _as the number of negative responses following a missing response, and *a*_11 _as the number of positive responses following a missing response. If *a*_10 _≤ *a*_11 _impute the value 1.

(e) In all the other cases impute a random draw from the empirical distribution of the dichotomous items, based on their proportion of positive responses.

#### Rasch model - RAS, RAI and RAS-R

The imputation by the Rasch model consists in susbituting the missing values using the rounded value of the probability of obtaining a positive response predicted by the Rasch model:(11)

In the RAS method,  is obtained by rounding *pnj*, and in the RAS-R method,  is randomly drawn from a Bernoulli distribution using *p_nj _*as its parameter.

These two methods are implemented in the OPLM software [24] to impute missing data in the One Parameter Logistic Model [25], of which the Rasch model is a particular case.

In the RAI (Iterative Rasch model) method, we substitute the missing data by the RAS model, and then reestimate the parameters of the Rasch model with the subsituted values leading to a second substitution. This process is repeated untill two successive iterations give exactly the same substituted values. The algorithm is generally stopped at the 10th iteration.

#### Summary table

Table 1 summarizes for each method whether it takes into account the ability of the individual, the difficulty of the item, the possibility of a random process or a likelihood based approach (when the imputation is based on a statistical model where the parameters are estimated by a maximum likelihood method).

**Table 1:** Summary table of the characteristics of the imputation methods used to handle missing data

Method	Ability of the individual	Difficulty of the item	Addition of a random process	Likelihood based approach
NOIMP				
LD				
WORST				
PMS	X		X	
IMS		X	X	
CIM	X	X	X	
ICS	X	X		
LOG	X	X	X	X
MOK	X	X		
RAS	X	X	X	X
RAI	X	X		X

#### Note on the imputation process

Imputation of missing data is only carried out for individuals having more than 50% non-missing data (at least 3 responses among the 5 items). This restriction is commonely used in practice, for example for the SF-36 and QLQC30 questionnaires [9,10] and Sijtsma and van der Ark [17] suggest that this yields more stable results. Note that for the analysis, the individuals with more than 3 missing items are not omitted but only their observed responses have been used.

For the 1000 simulated datasets, using this restriction, imputation could not be performed for an average of 6.0 individuals (over 500 individuals - ~ 1%) when *π *= 10%, of 37.3 individuals (over 500 individuals - ~7%) when *π *= 20% and of 97.2 individuals (over 500 individuals - ~ 19%) when *π *= 30%.

We note that with the ICS, LOG and LOG-R methods, imputation might not be possible in some cases:

*• *for ICS, if the most correlated item (of an item presenting a missing response) is also missing,

*• *for LOG(-R), if the logistic model used to fit a missing response includes covariates with missing values.

### Studied parameters

We evaluate the impact of the chosen method to handle missing dataon different parameters.

#### Scalability index

Loevinger's H coefficient [2] is used in non parametric Item Response Theory [3], and measures the scalability of a questionnaire. It can be defined as(12)

with *Cov*(*X_j_*, *X_k_*) the covariance between the items *j *and *k*, and *Cov*^(0)^(*X_j_*, *X_k_*) the maximum possible covariance between these two items with fixed marginal frequencies.

#### Parameters of the Rasch model

We studied the bias in different ways: the bias in estimating the  parameter, the bias in estimating the variance of the *δ_j _*parameters , and the bias in estimating the variance of the latent trait (*σ*^2^).

A positive bias on *v *for instance signifies that the latent trait is underestimated (or that the difficulty parameters of the items are globally overestimated) and corresponds to an optimistic result.

The variance  of the *δ *parameter is defined by

with  the mean on the 1000 replication of the estimations of the *δ_j _*parameters. A positive bias on this parameter signifies that the dispersion of the difficulty parameters is overestimated.

The variance parameter of the latent trait *σ*^2 ^represents the dispersion of the latent trait.

#### Fit of the Rasch model

In order to evaluate the impact of the imputation methods, we investigated the fit test statistic *Q*_1 _[4]. In this test, we compared for each score the positive responses to each item with the frequencies expected under the Rasch model assumption. Under the null assumption, the statistic follows a chi-square distribution. In this study, we evaluated on the 1000 replications of each case, the rate of rejection of the null assumption "Fit of the Rasch model". This estimation allows evaluating the type-I error of this fit test. It is expected that the rate of rejection of the null assumption will be close to 5% (because the former datasets are simulated with a Rasch model). If the 95% confidence interval does not contain the value 5%, the corresponding imputation method does not allow maintaining the type-I error to its expected level.

#### Reliability of the estimation of the latent trait

The Personal Separation Index (PSI) is a measure of the reliability of the scale. It can be computed as(13)

where  is evaluated by(14)

with  being the evaluated standard error of the estimation of the *θ_n _*parameter.

#### Biases on the parameters

For Loevinger's H coefficient (H) and Personal Separation Index (PSI), the biases in estimating these parameters are computed by comparing the estimation for each replication to the corresponding estimation obtained with complete datasets. For these two estimators, if Ψ is the random variable representing the estimator, we denote *ψ_l _*the estimation obtained for the *l*th replication and  the corresponding value with the full dataset. Then,(15)

For *ν*,  and *σ*^2^, the bias is computed by comparing the average of the estimations obtained on the 1000 replications to the values used in the simulation design (0 for *v*, 0.5 for  and 1 for *σ*^2^).

The bias is considered as negligible if it is lesser than 0.05 for *H *and *PSI*, lesser than 0.1 for *v *and lesser than 0.2 for . For *Q*_1_, the bias is considered as negligible if the 95% confidence interval of the rate of rejection of the assumption "*H*_0 _: fit of the Rasch Model" contains the value 5%. For *σ*^2^, the bias is considered as negligible if the estimation is included in the interval [0.71; 1.37] that contains 95% of the estimations of *σ*^2 ^obtained with the full datasets. Since the bias on *σ*^2 ^is computed as , it is considered as large if it is lesser than 0.71 - 1 = -0.29 or greater than 1.37 - 1 = 0.37, and small otherwise.

### Software

All analyses were done using Stata software. Loevinger's H was computed with the -loevh- command [26] (using the pairwise option), and the parameters of the Rasch model were estimated with -raschtest- [27] commands. The simulations were carried out with the -simirt- module. Three Stata modules (-imputeitems-, -imputerasch- and -imputemok-) were written to impute the missing data. All these Stata modules can be downloaded from the website of the first author http://www.anaqol.org.

## Results

The results given in this section are based on the mean results of the 1000 replications of each case. Formal statistical tests have been carried out to determine potential *π *and *ρ *effects for each imputation method on the bias of each studied parameter. In the event, all the tests were statistically significant, which raises the problematic issue of the distinction between statistically significant results and meaningfull results or results of practical importance. This is why, the above mentioned thresholds are proposed to help determine small and large bias.

The standard errors of the evaluations of all parameters have been computed, but, since they remained very stable whatever the values of *π*, *ρ *and the missing data mechanism (MCAR, MAR, MNAR), they were not included in the tables.

Tables 2 to 7 present respectively the bias in estimating the Loevinger's H coefficient (table 2), the *v *(table 3),  (table 4) and *σ*^2 ^(table 5) parameters, the rate of rejection of the Rasch model by the Q1 test (table 6) and the bias in estimating the PSI (table 7), for all the studied values of the *w*, *π *and *ρ *parameters.

**Table 2:** Bias on the Loevinger's H coefficient as a function of the rate of missing data per item (*π*), the value of the correlation coefficient *ρ *between the latent variable Θ and the propensity to have missing data *ξ *for each method for handling missing data (results for *w *= 0/*w *= 1)

	*π *= 10%			*π *= 20%			*π *= 30%		
Method	*ρ *= 0.0	*ρ *= -0.4	*ρ *= -0.9	*ρ *= 0.0	*ρ *= -0.4	*ρ *= -0.9	*ρ *= 0.0	*ρ *= -0.4	*ρ *= -0.9
PMS	0.08/0.08	0.08/0.08	0.08/0.07	0.14/0.13	0.14/0.13	0.14/0.13	0.18/0.17	0.18/0.17	0.17/0.16
PMS-R	0.04/0.04	0.04/0.04	0.04/0.04	0.07/0.08	0.07/0.07	0.07/0.07	0.10/0.10	0.09/0.09	0.09/0.09
IMS	-0.02/-0.02	-0.02/-0.02	-0.02/-0.02	-0.03/-0.03	-0.04/-0.04	-0.04/-0.04	-0.04/-0.04	-0.05/-0.05	-0.06/-0.06
IMS-R	-0.04/-0.04	-0.04/-0.04	-0.04/-0.04	-0.07/-0.07	-0.07/-0.07	-0.08/-0.08	-0.09/-0.09	-0.09/-0.09	-0.10/-0.10
CIM	0.09/0.09	0.09/0.09	0.09/0.09	0.15/0.15	0.15/0.15	0.15/0.15	0.19/0.19	0.20/0.20	0.19/0.20
CIM-R	0.05/0.04	0.05/0.04	0.05/0.04	0.08/0.07	0.09/0.07	0.08/0.07	0.11/0.09	0.11/0.09	0.10/0.08
ICS	0.04/0.04	0.04/0.04	0.04/0.04	0.07/0.07	0.07/0.07	0.07/0.07	0.09/0.09	0.09/0.09	0.08/0.08
LOG	0.03/0.04	0.03/0.04	0.03/0.03	0.04/0.04	0.04/0.04	0.03/0.03	0.04/0.03	0.03/0.03	0.00/0.01
LOG-R	-0.01/-0.01	-0.01/-0.01	-0.01/-0.01	-0.02/-0.02	-0.02/-0.03	-0.03/-0.03	-0.04/-0.04	-0.05/-0.05	-0.06/-0.06
MOK	0.05/0.05	0.05/0.05	0.05/0.05	0.09/0.10	0.09/0.09	0.09/0.09	0.12/0.12	0.12/0.12	0.11/0.12
RAS	0.02/0.02	0.02/0.02	0.02/0.02	0.04/0.04	0.04/0.04	0.03/0.04	0.04/0.05	0.04/0.05	0.04/0.05
RAS-R	-0.01/-0.01	-0.01/-0.01	-0.01/-0.01	-0.01/-0.01	-0.01/-0.01	-0.01/-0.01	-0.01/-0.02	-0.02/-0.02	-0.02/-0.02
RAI	0.03/0.03	0.03/0.03	0.03/0.03	0.12/0.10	0.12/0.10	0.11/0.10	0.16/0.15	0.16/0.15	0.15/0.15
WORST	-0.05/-0.04	-0.03/-0.03	-0.02/-0.01	-0.09/-0.07	-0.07/-0.05	-0.04/-0.02	-0.11/-0.09	-0.09/-0.07	-0.07/-0.04
NOIMP	0.00/-0.00	-0.00/-0.00	-0.00/-0.00	-0.00/-0.00	-0.00/-0.00	-0.00/-0.00	-0.00/0.00	-0.01/-0.01	-0.04/-0.04
LD	0.00/-0.00	0.00/-0.00	-0.00/-0.00	-0.00/0.00	-0.00/-0.00	-0.02/-0.01	0.00/0.00	-0.00/-0.00	-0.01/-0.01

**Table 3:** Bias on the *v *parameter as a function of the rate of missing data per item (*π*), the value of the correlation coefficient *ρ *between the latent variable Θ and the propensity to have missing data *ξ *for each method for handling missing data (results for *w *= 0/*w *= 1)

	*π *= 10%			*π *= 20%			*π *= 30%		
Method	*ρ *= 0.0	*ρ *= -0.4	*ρ *= -0.9	*ρ *= 0.0	*ρ *= -0.4	*ρ *= -0.9	*ρ *= 0.0	*ρ *= -0.4	*ρ *= -0.9
PMS	-0.04/-0.08	-0.04/-0.08	-0.03/-0.07	-0.04/-0.13	-0.04/-0.13	-0.05/-0.14	-0.05/-0.15	-0.08/-0.18	-0.11/-0.22
PMS-R	0.00/-0.03	-0.00/-0.03	-0.00/-0.03	0.00/-0.06	-0.00/-0.07	-0.03/-0.09	-0.00/-0.09	-0.03/-0.12	-0.07/-0.16
IMS	0.00/0.04	-0.03/0.02	-0.05/-0.00	0.00/0.08	-0.05/0.04	-0.12/-0.03	-0.00/0.13	-0.09/0.04	-0.19/-0.06
IMS-R	0.00/0.00	-0.02/-0.02	-0.04/-0.04	0.00/-0.00	-0.03/-0.03	-0.08/-0.08	-0.00/0.00	-0.06/-0.05	-0.13/-0.13
CIM	0.04/0.04	0.03/0.03	0.04/0.04	0.06/0.06	0.06/0.06	0.04/0.04	0.07/0.09	0.03/0.05	-0.00/0.00
CIM-R	0.02/0.04	0.01/0.03	0.01/0.04	0.03/0.01	0.01/0.00	0.01/-0.02	0.03/0.02	-0.01/-0.02	-0.05/-0.07
ICS	0.00/-0.02	-0.00/-0.03	-0.00/-0.03	0.00/-0.06	-0.01/-0.05	-0.03/-0.07	-0.00/-0.06	-0.03/-0.10	-0.07/-0.14
LOG	0.00/0.03	-0.02/0.01	-0.03/-0.01	0.00/0.05	-0.04/0.01	-0.11/-0.05	-0.00/0.09	-0.08/0.01	-0.19/-0.10
LOG-R	0.00/-0.00	-0.02/-0.01	-0.03/-0.03	0.00/-0.00	-0.03/-0.03	-0.08/-0.08	-0.00/0.00	-0.06/-0.06	-0.14/-0.13
MOK	-0.01/0.01	-0.02/0.00	-0.03/-0.00	-0.02/0.02	-0.04/0.01	-0.08/-0.02	-0.03/0.05	-0.08/0.00	-0.13/-0.05
RAS	0.00/-0.06	-0.00/-0.06	0.01/-0.06	0.00/-0.12	-0.00/-0.11	-0.00/-0.12	-0.00/-0.17	-0.01/-0.18	-0.02/-0.20
RAS-R	0.00/-0.04	-0.01/-0.05	-0.01/-0.05	0.00/-0.08	0.01/-0.09	-0.03/-0.11	-0.00/-0.12	-0.03/-0.14	-0.06/-0.17
RAI	0.00/-0.06	-0.00/-0.06	0.00/-0.06	0.00/-0.06	0.00/-0.06	-0.00/-0.07	-0.00/-0.08	-0.02/-0.11	-0.05/-0.15
WORST	0.23/0.23	0.22/0.20	0.21/0.19	0.38/0.36	0.36/0.34	0.33/0.31	0.45/0.44	0.41/0.40	0.36/0.35
NOIMP	0.00/0.00	-0.01/-0.01	-0.02/-0.02	0.00/-0.00	-0.02/-0.02	-0.06/-0.06	-0.00/0.00	-0.05/-0.04	-0.11/-0.11
LD	0.00/-0.00	-0.10/-0.08	-0.21/-0.19	0.00/-0.00	-0.20/-0.19	-0.45/-0.42	-0.00/-0.00	-0.31/-0.29	-0.70/-0.67
FC	0.00/-0.00	0.00/-0.00	-0.00/0.00	-0.00/-0.00	-0.00/0.00	0.00/0.00	-0.00/0.00	-0.00/0.00	0.00/0.00

**Table 4:** Bias on the variance of the *δ_j _*parameters as a function of the rate of missing data per item (*π*), the value of the correlation coefficient *ρ *between the latent variable Θ and the propensity to have missing data *ξ *for each method for handling missing data (results for *w *= 0/*w *= 1)

	*π *= 10%			*π *= 20%			*π *= 30%		
Method	*ρ *= 0.0	*ρ *= -0.4	*ρ *= -0.9	*ρ *= 0.0	*ρ *= -0.4	*ρ *= -0.9	*ρ *= 0.0	*ρ *= -0.4	*ρ *= -0.9
PMS	-0.05/-0.07	-0.05/-0.07	-0.06/-0.08	-0.09/-0.10	-0.09/-0.10	-0.09/-0.11	-0.10/-0.12	-0.10/-0.11	-0.09/-0.09
PMS-R	-0.08/-0.08	-0.08/-0.08	-0.08/-0.08	-0.13/-0.13	-0.13/-0.13	-0.13/-0.13	-0.16/-0.15	-0.15/-0.14	-0.15/-0.13
IMS	0.20/0.21	0.20/0.21	0.20/0.20	0.37/0.41	0.38/0.39	0.40/0.39	0.47/0.56	0.49/0.53	0.55/0.54
IMS-R	-0.03/-0.03	-0.03/-0.03	-0.03/-0.03	-0.05/-0.05	-0.05/-0.05	-0.04/-0.05	-0.06/-0.07	-0.06/-0.07	-0.03/-0.06
CIM	0.10/0.13	0.10/0.13	0.09/0.12	0.20/0.27	0.20/0.25	0.18/0.23	0.28/0.39	0.26/0.36	0.24/0.31
CIM-R	0.03/-0.03	0.02/-0.03	0.02/-0.02	0.05/0.06	0.05/0.05	0.03/0.03	0.07/0.07	0.06/0.06	0.06/0.04
ICS	-0.06/-0.06	-0.06/-0.06	-0.06/-0.06	-0.10/-0.09	-0.09/-0.09	-0.10/-0.09	-0.11/-0.11	-0.11/-0.10	-0.11/-0.10
LOG	0.16/0.16	0.16/0.16	0.16/0.15	0.28/0.31	0.29/0.29	0.30/0.28	0.38/0.43	0.40/0.40	0.44/0.37
LOG-R	-0.00/-0.00	-0.00/-0.00	-0.00/-0.00	-0.02/-0.01	-0.01/-0.02	-0.01/-0.02	-0.03/-0.03	-0.02/-0.03	0.00/-0.03
MOK	0.16/0.16	0.16/0.16	0.16/0.16	0.30/0.32	0.31/0.31	0.31/0.30	0.40/0.43	0.41/0.42	0.43/0.40
RAS	-0.22/-0.21	-0.23/-0.22	-0.22/-0.21	-0.34/-0.31	-0.34/-0.31	-0.34/-0.30	-0.39/-0.33	-0.39/-0.32	-0.40/-0.30
RAS-R	-0.18/-0.18	-0.18/-0.17	-0.18/-0.17	-0.28/-0.26	-0.28/-0.26	-0.28/-0.25	-0.33/-0.30	-0.32/-0.29	-0.32/-0.27
RAI	-0.22/-0.21	-0.22/-0.21	-0.22/-0.22	-0.19/-0.23	-0.19/-0.22	-0.20/-0.22	-0.19/-0.21	-0.19/-0.21	-0.20/-0.19
WORST	-0.01/0.08	-0.01/0.07	-0.01/0.08	0.11/0.26	0.09/0.25	0.06/0.22	0.20/0.43	0.13/0.37	0.06/0.30
NOIMP	0.00/0.00	0.00/0.00	0.00/0.00	0.00/0.00	0.01/0.01	0.01/0.00	0.01/0.00	0.01/0.00	0.03/0.00
LD	0.00/0.00	0.02/0.02	0.09/0.08	0.00/0.00	0.09/0.08	0.42/0.37	0.01/0.02	0.21/0.19	0.98/0.92
FC	0.00/0.00	0.00/0.00	0.00/0.00	0.00/0.00	0.00/0.00	0.00/0.00	0.00/0.00	0.00/0.00	0.00/-0.00

**Table 5:** Bias on the *σ*^2 ^parameters as a function of the rate of missing data per item (*π*), the value of the correlation coefficient *ρ *between the latent variable Θ and the propensity to have missing data *ξ *for each method for handling missing data (results for *w *= 0/*w *= 1)

	*π *= 10%			*π *= 20%			*π *= 30%		
Method	*ρ *= 0.0	*ρ *= -0.4	*ρ *= -0.9	*ρ *= 0.0	*ρ *= -0.4	*ρ *= -0.9	*ρ *= 0.0	*ρ *= -0.4	*ρ *= -0.9
PMS	0.61/0.58	0.61/0.58	0.62/0.57	1.27/1.14	1.27/1.14	1.26/1.11	1.64/1.58	1.59/1.57	1.56/1.50
PMS-R	0.34/0.35	0.34/0.35	0.34/0.34	0.62/0.64	0.62/0.62	0.61/0.62	0.85/0.86	0.82/0.86	0.79/0.81
IMS	-0.15/-0.14	-0.15/-0.15	-0.15/-0.16	-0.24/-0.24	-0.24/-0.24	-0.27/-0.27	-0.28/-0.29	-0.30/-0.29	-0.34/-0.32
IMS-R	-0.23/-0.22	-0.23/-0.23	-0.22/-0.23	-0.38/-0.38	-0.37/-0.38	-0.38/-0.39	-0.45/-0.47	-0.46/-0.46	-0.46/-0.48
CIM	0.59/0.57	0.60/0.58	0.62/0.58	1.15/1.09	1.17/1.13	1.20/1.14	1.54/1.48	1.55/1.56	1.55/1.60
CIM-R	0.32/0.28	0.33/0.29	0.35/0.30	0.59/0.50	0.61/0.52	0.62/0.52	0.80/0.64	0.78/0.66	0.78/0.65
ICS	0.32/0.33	0.32/0.33	0.33/0.33	0.57/0.59	0.57/0.61	0.56/0.57	0.76/0.77	0.74/0.78	0.71/0.74
LOG	0.19/0.20	0.19/0.20	0.18/0.18	0.22/0.22	0.22/0.24	0.16/0.19	0.18/0.16	0.13/0.18	0.02/0.07
LOG-R	-0.03/-0.13	-0.03/-0.03	-0.03/-0.04	-0.12/-0.12	-0.13/-0.12	-0.15/-0.15	-0.20/-0.23	-0.22/-0.22	-0.27/-0.29
MOK	0.30/0.31	0.30/0.31	0.30/0.29	0.54/0.56	0.55/0.57	0.54/0.55	0.75/0.74	0.72/0.76	0.69/0.73
RAS	0.26/0.28	0.26/0.28	0.28/0.27	0.49/0.50	0.49/0.53	0.49/0.53	0.65/0.67	0.62/0.70	0.60/0.70
RAS-R	0.04/0.05	0.04/0.05	0.05/0.04	0.06/0.07	0.07/0.09	0.07/0.09	0.10/0.09	0.08/0.10	0.07/0.09
RAI	0.29/0.31	0.29/0.31	0.30/0.30	1.12/0.92	1.12/0.95	1.11/0.95	1.53/1.40	1.49/1.42	1.46/1.41
WORST	-0.25/-0.21	-0.16/-0.13	-0.04/-0.03	-0.42/-0.27	-0.30/-0.25	-0.15/-0.11	-0.50/-0.47	-0.42/-0.36	-0.28/-0.14
NOIMP	0.01/0.02	0.01/0.02	0.02/0.02	0.00/0.01	0.01/0.02	0.00/-0.00	0.02/0.00	-0.00/0.01	-0.01/-0.01
LD	0.01/0.02	0.02/0.02	-0.00/-0.01	0.02/0.02	0.01/0.02	-0.05/-0.04	0.03/0.03	-0.02/0.02	-0.14/-0.12
FC	0.01/0.01	0.01/0.01	0.02/0.01	0.00/0.00	0.01/0.02	0.01/0.01	0.01/0.00	0.00/0.01	0.01/0.01

**Table 6:** Rate of rejection of the Rasch model assumption with the Q1 test as a function of the rate of missing data per item (*π*), the value of the correlation coefficient *ρ *between the latent variable Θ and the propensity to have missing data *ξ *for each method for handling missing data (results for *w *= 0/*w *= 1) [*: values significantly different of 5%]

	*π *= 10%			*π *= 20%			*π *= 30%		
Method	*ρ *= 0.0	*ρ *= -0.4	*ρ *= -0.9	*ρ *= 0.0	*ρ *= -0.4	*ρ *= -0.9	*ρ *= 0.0	*ρ *= -0.4	*ρ *= -0.9
PMS	5.2/6.5	5.2/6.6*	4.9/6.3*	5.7/9.2*	5.9/9.7*	6.2/8.1*	4.6/22.3*	6.3/17.6*	6.2/18.5*
PMS-R	5.6/5.2	6.0/5.6	4.0/4.7	3.9/8.0*	4.3/6.4	6.2/6.0	5.2/8.5*	5.9/8.3*	6.7*/8.2*
IMS	5.6/3.9	5.4/4.6	6.0/5.8	4.5/4.6	6.3/4.4	6.1/5.3	3.9/4.5	3.9/5.0	6.1/5.8
IMS-R	6.0/6.0	6.0/6.1	4.5/5.0	6.2/6.7*	4.4/5.7	4.9/5.1	4.7/7.4*	5.9/5.5	4.3/6.8
CIM	11.4*/9.7*	8.5*/9.3*	9.0*/8.2*	19.6*/19.5*	15.5*/18.5*	14.2*/19.1*	22.9*/29.0*	22.2*/28.8*	23.6*/32.7*
CIM-R	5.9/3.7	4.9/4.4	5.2/4.4	6.9*/4.4	6.9*/5.6	6.6*/4.5	6.9*/5.5	6.8/4.9	8.0*/6.3
ICS	49.6*/54.8*	51.5*/55.2*	51.7*/54.0*	85.0*/87.9*	86.0*/88.6*	85.0*/89.0*	94.1*/97.6*	93.7*/96.5*	95.6*/97.7*
LOG	33.0*/37.3*	34.9*/34.6*	35.8*/37.2*	64.2*/63.6*	63.2*/67.4*	61.5*/65.9*	65.5*/65.6*	63.2*/68.1*	60.4*/66.9*
LOG-R	10.6*/12.6*	13.1*/11.1*	11.9*/11.6*	19.1*/19.8*	19.5*/18.7*	17.7*/19.0*	22.2*/26.4*	24.5*/24.8*	22.6*/24.0*
MOK	16.1*/17.3*	17.9*/18.6*	19.2*/19.4*	42.2*/51.3*	44.3*/54.2*	49.4*/56.9*	58.5*/72.4*	60.2*/75.0*	67.8*/82.0*
RAS	21.8*/23.8*	22.8*/24.3*	21.5*/26.1*	61.2*/69.9*	63.9*/67.6*	64.2*/67.5*	80.6*/87.7*	83.3*/88.7*	81.0*/86.3*
RAS-R	4.7/4.9	5.9/5.3	6.2/6.2	6.0/6.2	6.9*/6.9*	7.3*/6.0	6.6*/6.6*	6.7*/16.9*	6.8*/5.7
RAI	21.9*/24.3*	22.9*/24.3*	22.6*/27.5*	17.8*/45.0*	19.6*/40.2*	18.3*/36.9*	10.1*/61.0*	14.1*/52.5*	15.4*/45.0*
WORST	6.8*/5.5	6.1/5.8*	5.1/3.2*	6.7*/6.9*	6.2/4.4	5.0/4.8	10.0*/6.9*	9.2*/5.8	8.0*/4.0
NOIMP	6.5/7.8*	7.5*/8.2*	6.9*/7.6*	9.2*/9.7*	11.0*/10.9*	10.2*/9.4*	13.3*/14.8*	13.8*/14.0*	9.6*/9.5*
LD	4.9/5.1	4.0/5.2	5.1/4.9	4.8/5.2	5.1/5.0	4.3/4.4	3.4*/3.3*	3.4*/4.5	3.3*/2.5*
FC	5.2/5.5	4.8/4.4	5.1/5.0	5.4/4.6	4.1/3.9	4.9/4.9	4.0/4.8	4.7/4.5	4.9/3.8*

**Table 7:** Bias on the PSI as a function of the rate of missing data per item (*π*), the value of the correlation coefficient *ρ *between the latent variable Θ and the propensity to have missing data *ξ *for each method for handling missing data (results for *w *= 0/*w *= 1)

	*π *= 10%			*π *= 20%			*π *= 30%		
Method	*ρ *= 0.0	*ρ *= -0.4	*ρ *= -0.9	*ρ *= 0.0	*ρ *= -0.4	*ρ *= -0.9	*ρ *= 0.0	*ρ *= -0.4	*ρ *= -0.9
PMS	0.09/0.09	0.09/0.09	0.09/0.10	0.13/0.13	0.13/0.13	0.13/0.13	0.13/0.09	0.13/0.09	0.12/0.09
PMS-R	0.06/0.06	0.06/0.06	0.06/0.06	0.08/0.08	0.08/0.08	0.08/0.08	0.07/0.06	0.07/0.06	0.06/0.06
IMS	-0.05/-0.05	-0.05/-0.05	-0.05/-0.05	-0.09/-0.09	-0.10/-0.09	-0.10/-0.10	-0.14/-0.05	-0.14/-0.05	-0.16/-0.05
IMS-R	-0.06/-0.06	-0.06/-0.06	-0.06/-0.06	-0.12/-0.12	-0.12/-0.12	-0.12/-0.12	-0.17/-0.06	-0.17/-0.06	-0.18/-0.06
CIM	0.09/0.08	0.09/0.09	0.09/0.09	0.12/0.12	0.12/0.12	0.12/0.12	0.12/0.08	0.12/0.09	0.12/0.09
CIM-R	0.05/0.05	0.05/0.05	0.06/0.05	0.07/0.06	0.08/0.06	0.08/0.07	0.06/0.05	0.06/0.05	0.06/0.05
ICS	0.05/0.05	0.05/0.05	0.05/0.05	0.07/0.07	0.07/0.07	0.07/0.07	0.05/0.05	0.05/0.05	0.04/0.05
LOG	0.02/0.02	0.02/0.02	0.02/0.02	-0.00/0.00	-0.00/0.00	-0.01/-0.01	-0.05/0.02	-0.06/0.02	-0.08/0.02
LOG-R	-0.02/-0.02	-0.02/-0.02	-0.02/-0.02	-0.06/-0.06	-0.06/-0.06	-0.07/-0.05	-0.11/-0.02	-0.11/-0.02	-0.13/-0.02
MOK	0.05/0.05	0.05/0.05	0.04/0.04	0.06/0.07	0.06/0.06	0.06/0.06	0.05/0.05	0.05/0.05	0.04/0.04
RAS	0.05/0.05	0.05/0.05	0.05/0.05	0.07/0.07	0.07/0.07	0.07/0.07	0.05/0.06	0.05/0.06	0.04/0.06
RAS-R	0.01/0.01	0.01/0.01	0.01/0.01	-0.00/0.00	0.00/0.00	0.00/0.00	-0.03/0.01	-0.03/0.01	-0.03/0.01
RAI	0.05/0.01	0.05/0.06	0.05/0.06	0.12/0.12	0.12/0.12	0.12/0.12	0.13/0.06	0.12/0.06	0.12/0.06
WORST	-0.06/-0.06	-0.04/-0.04	-0.01/-0.01	-0.13/-0.12	-0.09/-0.09	-0.05/-0.05	-0.19/-0.06	-0.16/-0.04	-0.12/-0.01
NOIMP	-0.03/-0.03	-0.03/-0.03	-0.03/-0.03	-0.06/-0.06	-0.07/-0.06	-0.07/-0.06	-0.10/-0.03	-0.10/-0.03	-0.11/-0.03
LD	-0.00/-0.00	-0.00/-0.00	-0.01/-0.00	-0.00/-0.00	-0.01/-0.01	-0.03/-0.02	-0.01/-0.00	-0.02/-0.00	-0.07/-0.01

### MCAR and MAR cases

Bias is encountered for all methods in the MCAR (*w *= 0 and *ρ *= 0) and MAR (*w *= 1, *ρ *= 0) cases, but to a different extent. For all the methods and all the studied parameters, the bias increases with *π*, although for some methods, the bias can be small even for high values of *π*.

With the exception of IMS and LOG, all the methods that do not incorporate a random process (PMS, ICS, CIM, MOK, RAS, RAI, WORST) present bias on the majority of the parameters (at least 3 among the 6 studied parameters) in these two cases. IMS presents small bias in the MCAR case (only for  and PSI), but is more biased in the MAR case. This result could be expected because IMS is the only imputation method (with WORST) that does not incorporate the difficulty of the items in the imputation process.

If the methods using a random process are generally better than the similar methods with no random process, only LOG(-R), RAS-R, NOIMP and LD present few bias on the majority of the parameters in the MCAR and MAR cases. For these methods, we note a higher rate of rejection of the Rasch model than exepcted, a bias on  (for LOG(-R) and RAS-R), or on the PSI (for LOG and NOIMP). LD is the only method that dispays a rate of rejection of the Rasch model which is significantly lesser than 5%. This phenomenon can be explained by the fact that LD omits all the individuals with at least one missing value, and consequently, the number of remaining individuals is smaller as compared to the others methods. As a consequence, the *Q*1 test, which is a chi-square type test, might lack power to detect small deviations to the Rasch model.

On the opposite, MOK, CIM and WORST present a relevant bias on all the parameters except *ν *in the MCAR and MAR cases, and PMS, RAS and RAI are very biased methods in the MAR case.

### MNAR cases

All the methods present several bias in the MNAR case (*ρ *≠ 0). For all the methods and all the studied parameters, the bias increases with *π*, even if for some methods, the bias can be negligible even for high values of *π*. Generally, the effect of the *ρ *parameter is smaller (except for WORST or LD) and can reinforce or reduce the bias when *ρ *increases in absolute value.

NOIMP, LOG-R, and RAS-R are the three methods that produce the smallest number of biased parameters in the MNAR case. Indeed, if the rate of missing value is weak (*π *= 10%), RAS-R is unbiased on all the studied parameters, and LOG-R and NOIMP are biased only on the rate of rejection of the Rasch model. Neverthless, when the rate of missing value is larger than 10%, these three methods present bias on the rate of rejection of the Rasch model, NOIMP and LOG-R present bias on *v *and PSI, and RAS-R present bias on .

For the methods PMS, IMS, CIM, LOG and RAS, the addition of a random process in the imputation process seems to reduce the bias on all the parameters. As for the MCAR and MAR cases, LD is the only method that produces a too lower rate of rejection of the Rasch model than expected, and this could be explained by the number of individuals used with this method. WORST and RAI produce a systematic relevant bias on all the studied parameters, and PMS, CIM, MOK and RAS displaya relevant bias on 5 of the 6 studied parameters.

## Discussion

Sixteen methods for handling missing data have been investigated in the framework of psychometric validation of a PRO scale using IRT-based methodology. Several situations were considered according to the type of missing data one might encounter in practice: namely MCAR, MAR or MNAR type of missing data.

Some of the investigated methods can be referred to as principled methods, mostly relying on likelihood-based analysis, such as Rasch models or on an handling of the missing data without imputation, such as NOIMP or LD and others as unprincipled or ad-hoc methods such as PMS, IMS, CIM or WORST. Some of the latter methods (PMS, IMS) are frequently used for missing data imputation in HR-QoL scales even though they are known to provide biased estimations [28] in cross-sectional or longitudinal settings. By contrast, the former principled methods are likely to be consistent under MCAR and sometimes MAR mechanisms.

As expected, we observed that principled methods such as NOIMP and LD were rarely biased (except regarding the Q1 test) under MCAR and MAR mechanisms whatever the amount of missing data. By contrast, unprincipled methods such as PMS, CIM, ICS, MOK or WORST were almost systematically biased even under MCAR and MAR mechanisms. More precisely, most of the methods taking into account the ability of the individuals in the imputation process tend to overestimate the psychometric quality of the scale (measured for example by the Loevinger's H coefficient or the PSI). This result was already noted by Huisman [16] and reflects the fact that these methods assume good properties of the scale and hence, tend to incorrectly enhance its psychometric performance during imputation.

Moreover, the methods incorporating the ability of the individual also overestimated the variance of the latent trait (*σ*^2^) thus creating artificial heterogeneity between the individuals. As a matter of fact, such methods will more likely impute a negative (positive) response to a patient who's observed score is low (high) and consequently falsely amplify the distance between individuals on the latent trait scale. In most cases, the addition of a random process helped to diminish the bias quite importantly and should be systematically used when possible [29].

The impact of the imputation methods in terms of bias was usually intensified under MNAR mechanism except for NOIMP, LOG-R and RAS-R that displayed the most robust results and remained usually unbiased (bias, if present, remained rather slight when *π <*20%). However, this time, LD was also affected and displayed bias, especially on the *ν *and item difficulties variance parameters . Moreover, the type I error of the goodness-of-fit Q1 test was underestimated for LD when the amount of missingness was high (*π *= 30%), possibly reflecting a loss in power. It is well known that MNAR missing data may importantly affect the representativeness of a study sample in relation to the target population. In this study, MNAR missing data were simulated such as patients with lower level on the latent trait (reduced HR-QoL for instance) had a higher non response propensity. The likelihood of missing data could also be larger as the item difficulties increased. As a consequence, in case of MNAR data, the data suffer from sample selection bias: for instance, patients having the highest levels on the latent trait primarily remained in the study and, under some circumstances, the easiest items were more often answered to. This leads to usually overestimate the latent trait level (and jointly underestimate item difficulties) producing negative bias for the *v *parameter except for the WORST method that systematically underestimates the latent trait level by only imputing negative responses. A *ρ *effect was observed for most of the methods on *v *(except for CIM(-R)) and it could sometimes be quite large. This effect, reflecting the strong informativity of the missing data, generally enlarged the bias that was already observed except for the WORST method for which the bias was attenuated but still remained.

Although one could expect poor results using such unprincipled or ad-hoc simple imputation methods for handling missing data, little was known about the impact of using one method or another on the quality of questionnaire validation studies. Indeed, missing data are solely described in such studies for assessing acceptability of a questionnaire [30,31] and PMS or IMS-based methods are often used for imputation. As a matter of fact, one of the most commonly used imputation method in a wide range of PRO studies (validation or clinical research studies), namely PMS, displayed poor properties regarding bias on a large number of parameters whatever the studied situation (MCAR, MAR or MNAR data) and the amount of missing data. As a consequence, this method should be avoided because it is very likely to overestimate the psychometric qualities of scales. Furthermore, PMS might also decrease the power of a test aimed at comparing two groups of patients on a PRO measure by artificially increasing the variance of the latent trait. This is in line with other authors such as Chavance [22] who recommends the use of this imputation method only if the rate of missing values is small (inferior to 5%). Moreover, Fayers et al. [32] gave six conditions for using PMS, which are rarely present from a practical point of view.

The methods based on Rasch models without a random process (RAS and RAI) often displayed poor results regarding bias on several parameters, especially on the variance of the latent trait that was overestimated along with the dispersion of the parameters difficulties that was underestimated. It was unforeseen that these possibly attractive methods should in fact be avoided, even though it was already noted, but not formally evaluated by Sijtsma and van der Ark [17].

The analysis without imputation NOIMP is a good alternative to simple imputation, provided all the responses are used in the analysis, under MCAR, MAR and even MNAR data. This result could be expected because one of the most important properties of the Rasch model is the specific objectivity. This property yields that i) all estimated difficulty parameters are independent of the sample used for estimation (item parameter invariance), ii) all latent trait related parameters are also independent of the items used for estimation (person parameter invariance). Consequently, the estimations of the parameters are consistent, even with an incomplete dataset, and whatever the type of missing data. However some specificities of this study have to be mentioned: Loevinger's H coefficient has been computed by pairwise technique which consists in using all the contingency tables between each pair of items in order to compute this indice (the usual procedure consists in estimating this indice by listwise deletion). The same remark can be made concerning the parameters of the Rasch model that have been estimated by marginal maximum likelihood allowing taking into account all observed responses. Other methods of estimation (conditional maximum likelihood for example), omitting the individuals with one or several missing data, might end to poorer results.

Our study focused on simple imputation methods that are frequently encountered in practice in most studies aiming at validating or analysing PRO data. An important issue with such methods is that they will often lead to a misleading estimation of precision, which is often overestimated. Since our major objective was to highlight the strong deleterious impact that these methods also have in the framework of studies aiming at validating PRO scales, other alternative for handling missing data were not evaluated. This is the case of hot deck substitution [16,33], imputation based on the Response Function Imputation [17], and Two-way imputation [17,34]. Moreover, we have not tested multiple imputations methods, which are recommended by several authors [8,15,17], in order to provide valid inferences for statistical estimates from incomplete data and more stable results. However, under MCAR or MAR, multiple imputations should lead to analyses that are similar to likelihood based analyses, being asymptotically equivalent as the number of imputations increase.

## Conclusion

This study shows that the choice of the imputation method must be made with attention during the validation of a scale by a Rasch model in presence of missing data. If the missing data are suspected to be MCAR or MAR, several principled methods could be used, like RAS-R, NOIMP or LD methods. However, if the missing data are suspected to be MNAR, RAS-R or NOIMP might be preferred (and LD must be avoided), but it seems sensible to realize the analysis only if a small number of missing data (*π *= 10%) is present. If the number of missing data is too large, none of the methods used to handling missing data seems to produce accurate results on the majority of the parameters, and consequently, all the analyses might be biased. One can also stress that all the methods not including a random process, in particular PMS (that is the most popular method), should be disregarded.

Finally, the impact of the choice of an imputation method on the statistical properties of tests aimed at comparing PRO data from two groups of patients is also an important topic for future research and deserves investigation.

## Competing interests

The authors declare that they have no competing interests.

## Authors' contributions

JBH have made substantial contributions to conception and design, acquisition of data, analysis and interpretation of data. VS have made substantial contributions to conception and design, analysis and interpretation of data. RC have made substantial contributions to interpretation of data. All authors read and approved the final manuscript.

## Pre-publication history

The pre-publication history for this paper can be accessed here:

http://www.biomedcentral.com/1471-2288/11/105/prepub
